# DualSightNet: A novel dual architecture for visual quality control of railway infrastructure

**DOI:** 10.1371/journal.pone.0340789

**Published:** 2026-01-23

**Authors:** Christopher Mai, Luca Eisentraut, Jonas Ketterer, Ricardo Buettner

**Affiliations:** Chair of Hybrid Intelligence, Helmut-Schmidt-University/University of the Federal Armed Forces Hamburg, Hamburg, Germany; Najran University College of Computer Science and Information Systems, SAUDI ARABIA

## Abstract

To ensure the operational safety of trains, it is essential to monitor the condition of the rails. In order to detect problems ranging from wear and tear to possible sabotage, no comprehensive and continuous monitoring is carried out today. The objective of this study is to develop and robustly validate a deep-learning model capable of reliably classifying a broad range of safety-critical rail surface defects. The use of deep learning to classify defect types based on optical images is a promising approach, but the existing literature does not yet achieve a robust, high-performing classification for a broad range of failure types. Many approaches rely mainly on local feature extraction, but this carries the risk of overlooking global relationships, which are crucial for distinguishing certain defect types. This study addresses this gap by utilizing the specific features of this problem domain (small, local, and global defect types). To this end, DualSightNet is introduced as a hybrid architecture enhanced by an attention module for classifying a broad range of railway track surface defects. The model achieves a five-fold cross-validated average balanced accuracy of 97.55 % on a peer-reviewed, real-world dataset of 5,153 images covering seven defect types, recorded directly from an inspection vehicle under operational conditions, indicating strong generalization across the diverse real-world variations represented in the dataset. Compared to existing CNN- or Transformer-based approaches, DualSightNet is the first approach that combines local and global feature extraction through a gating-based fusion mechanism and then enhances the fused representation using an attention module, which enables substantially more robust multiclass defect recognition. This sets a new benchmark for our problem domain, surpassing previous approaches, which either lack broad defect coverage or do not employ rigorous cross-validated evaluation. Our results have far-reaching practical implications, proving that by leveraging problem-specific features, neural networks are able to robustly classify a broad range of defect types. The inference time of the proposed system (3.00 ms per image) makes DualSightNet suitable for deployment in automated inspection vehicles and real-time monitoring scenarios.

## Introduction

Each year, billions of passengers travel by train, making railway systems one of the most important means of transportation [1]. At the same time, the safety of this mode of transportation is directly linked to the condition of the rails, as trains, unlike motor vehicles, usually have no way of swerving and their braking distances are long [2,3]. Even small defects in the extensive rail network can therefore lead to consequences, including the derailment of the train. Monitoring the condition of the rail infrastructure continuously and comprehensively is therefore a relevant problem, but one that is currently often solved manually, only on a section-by-section basis or by means of occasional inspection runs. Close-meshed monitoring to detect wear and tear or even deliberate acts of sabotage is currently not possible, but would be highly relevant. An automated way of condition monitoring is therefore necessary [4].

The key for such an approach lies in the use of deep learning-based convolutional neural networks (CNN), which have experienced strong performance gains in recent years [5] and match or even exceed human performance [6] in many domains. Such methods are already widely used today, particularly in the field of automated quality control [7] and defect detection. In addition to training such a network from scratch, transfer learning is often used, in which a deep architecture is pre-trained on a very large, generalized image dataset and then re-trained on the actual problem [8]. In this way, deep architectures can be used, and the problem of overfitting [9] can be avoided at the same time. To achieve maximum performance, researchers employ deeper networks, hybrid architectures, and innovative feature fusion strategies, although these typically increase model complexity. Other approaches rely on the use of pre-processing filters [10].

Deep learning approaches have also been widely used to address the problem of railway quality control. While one group of studies focuses on object detection or on segmentation [11–13], other authors classify defects into defect types. The former approach is particularly relevant from a micro perspective, for example, if a repair is to be carried out automatically. For the macro perspective of area-wide monitoring, this is less decisive, as only the information about the type of defect and thus the decision as to whether a train must be stopped as a result is important. Currently, all relevant works addressing this task either do not cover a broad range of defect types or are not cross-validated.

Ackigoz/Korkmaz [19] developed a multi-scale residual convolutional network to distinguish between four classes: healthy, joints, squats, and severe squats, so-called “SSquats”. They expand the original dataset from 1,838 to 12,866 images with data augmentation. For evaluation, no cross-validation was performed. Instead, the authors used an 85%/15% train–test split, reporting an accuracy of 99.83%. Dang et al. [14] used non-optical sensors and ultrasonic waves to distinguish intact rails from those with cracks or bumps. Based on 900 samples, they classify three defect types and report an accuracy of 98.00% with a custom CNN (no broad coverage, non-cross-validated). Aydin et al. [15] use contrast-based image preprocessing and a lightweight CNN for feature extraction to reliably classify three fastener states (healthy, damaged, missing). A cubic SVM is used as the classifier, achieving an accuracy of 99.7% using 5-fold cross-validation. The study by Hu et al. [16] developed a two-stage hierarchical classification model for ultrasound B-scan images, identifying EfficientNet-B7 as the best backbone and using a total of eight different degrees of rail defect (four main types). The approach achieves an overall accuracy of 88.56%. Jatoi et al. [17] use the same dataset as the present study (seven classes). They tested five pre-trained models, such as Vision Transformer, DenseNet121, ResNet50, EfficientNet, and MobileNetV3. Transfer learning and fine-tuning were used for training, but no cross-validation was performed. Using a train-test split, DenseNet121 achieves the highest performance with an accuracy of 92.80%.

It can be seen that previous studies in this field have focused primarily on pure CNN approaches, meaning that hybrid architectures for this application have scarcely been explored so far. Moreover, the datasets used lack sufficient diversity in defect types, which limits the generalizability of existing methods, as various defect categories occur in real-world settings. In addition, most studies do not employ cross-validation to validate their models, which significantly weakens the robustness of the reported results, since single-split evaluations are not sufficiently reliable for a safety-critical application and carry the risk of a “lucky split” that can distort performance.

For reliable large-scale rail monitoring, visual systems must be able to classify all relevant defect types under realistic operating conditions. Current methods either focus on a small subset of defects, lack the necessary feature representation capacity to capture both local and global patterns due to their chosen architecture, or are insufficiently validated. This underscores the need for a model that can reliably detect defects despite their varying shapes and sizes while delivering robust performance.

Therefore, a **highly relevant research gap** emerges: to pave the way for reliable large-scale railway quality control, a model must be developed that classifies all relevant defect types and is rigorously validated using 5-fold cross-validation.

Our study aims to address this gap by proposing a model that takes into account the specific characteristics of railway defects. Those typically display both local, fine-grained defects, such as small cracks or flakings, and larger, more global defects, such as joints. Leveraging this aspect, we employ DualSightNet, a hybrid architecture consisting of a ResNet50 branch to address local patterns and a Swin Transformer V2 to utilize global context. This is enhanced by an Efficient Channel Attention (ECA) module, which further highlights the most important feature channels, thereby enabling the model to classify defects more robustly. To validate our approach, we utilize a peer-reviewed and comprehensive dataset of 5,153 images [18] and employ a 5-fold cross-validation. This dataset was selected because its seven defect classes cover a large portion of the defect types that occur in real-world railway operations. Furthermore, the images were captured under genuine environmental conditions, with cameras mounted on an inspection vehicle. The images contain varying levels of noise, shadowed regions, and foreign objects in the ballast bed, reflecting realistic operational conditions and thereby supporting the model’s ability to generalize. DualSightNet achieves an average balanced accuracy of 97.55%, setting a new benchmark for this problem domain. Our approach builds on two backbone models whose outputs are fused through a gating mechanism to achieve high balanced accuracy, which is essential in safety-critical environments. Despite this hybrid architecture, DualSightNet maintains a low inference time of 3.00 ms per image, making it suitable for deployment in automated inspection vehicles and real-time monitoring scenarios. Based on these results, our work has three contributions:

1. The results demonstrate that DualSightNet, which utilizes both local and global features, sets a new benchmark for quality control in the railway sector with an average balanced accuracy of 97.55%.
2. The proposed architecture shows robust classification performance across all relevant types of defects, even for strongly underrepresented classes.
3. The study illustrates that the simultaneous use of CNNs and Swin Transformers can set new benchmarks for classifying images.

This paper is structured as follows: After this introduction, the related work is presented. This is followed by the methodology, which introduces the architecture and training process. Finally, the results, interpretation of the results, practical implications, limitations and future research are presented.

## Related work

### Surface defects in railway systems

An intact and reliable rail network is essential for the safe operation of rail vehicles. Surface defects on the rails pose a serious risk to operational safety. In daily use, rails are subject to continuous wear, which in the long term leads to rolling contact fatigue (RCF). This can initially cause microscopic cracks to form, which, if undetected, can later develop into characteristic defects. Early detection of this damage is therefore crucial for safe operation. In this study, we examine seven different defect classes that were collected under real-world conditions in 2024 [18].

1. **Cracks:** Cyclic loading causes microcracks to form on or below the surface, which spread over time and can lead to other types of defects.
2. **Flakings:** Sidewall deformation occurs on the tread, typically caused by high lateral forces in tight corners.
3. **Grooves:** Longitudinal grooves along the rail, usually caused by abrasive wear or faulty grinding work.
4. **Joints:** Irregularities at rail joints or weld seams, often caused by poor connections or thermal stresses.
5. **Shellings:** Material chips off the surface when subsurface cracks propagate upward, typically caused by rolling contact fatigue.
6. **Spallings:** Larger surface breakouts form when multiple shelling cracks merge, usually as a result of advanced fatigue damage.
7. **Squats:** are localized depressions with a typical crack pattern that are often caused by dynamic wheel forces, such as imbalances.

Not every type of defect has the same impact on the condition of the rail infrastructure. While grooves or flaking are considered largely tolerable in the literature, depending on their location and severity, cracks or squats are considered an acute safety risk and require immediate repair. Shelling and spalling usually indicate advanced damage caused by RCF and require treatment in the medium term. Joints are considered potential weak points and should also be checked regularly. Therefore, a precise classification of defect types across all classes is crucial for condition-based maintenance of rail networks. Existing conventional testing methods, such as visual or ultrasonic inspection, are time-consuming and costly, and can therefore only be used selectively. Automated, image-based fault classification can be used more widely and can therefore help to detect critical faults at an early stage and initiate targeted measures.

### Deep learning for railway surface defect detection

Due to deep learning’s ability to classify images and their context at a high level, deep learning-based approaches have also been used for the assessment of railway quality for several years. A distinction can be made between segmentation or object recognition approaches and those that classify images. While the former primarily detects the position of the anomaly to be recognized, the latter approaches classify the image shown into predefined classes. Segmentation and object detection approaches, therefore, provide information about the type of damage as well as its (pixel-precise) position, which is a more difficult task and therefore tends to result in lower performance. One of the most effective approaches in this first group is the work of Haroon et al. [11], who perform object detection based on 3,500 images. Taking five classes (e.g., defective fasteners and fishplates) into account, the authors achieve a mean average precision of 95%. Zhang et al. [13] go one step further and use eight different classes and 400 samples. The authors, who also perform object detection, achieve a mean average precision (@0.5) of 85.6% [13]. The approach with the most diverse dataset from the group of studies performing object recognition or segmentation is chosen by Zhao et al. [12] in 2024, which includes 15 different defect types based on 5,557 samples. Based on this, they achieve a mean average precision (@0.5) of 88.1%.

Nevertheless, there is a conceptual problem: It is questionable whether the additional information provided by object detection or segmentation regarding the (pixel-precise) localization of the error is significant enough for the actual real-world problem that the resulting trade-off in terms of model performance is worth this additional information. This is not necessarily the case: as long as it is not yet possible to automatically correct the detected errors, knowing the exact location of the error is less important than detecting the error as accurately as possible. Therefore, the focus should be on high-performance and robust classification of rail images. To achieve this, two aspects are important: first, rails can exhibit a variety of error patterns. The developed model must therefore cover as many relevant error types as possible in order to avoid the risk of misclassifications due to error patterns not provided for in the architecture. Second, in such safety-critical applications, it is particularly important that the developed model achieves the reported performance robustly and methodically at the highest level, which is only possible through stratified k-fold cross-validation. Table 1 therefore provides an overview of the literature relevant to this paper, its coverage of the range of defect types, and whether it employs a cross-validated approach.

**Table 1:** Comparison of relevant classification literature on rail surface defect detection regarding coverage of five or more defect types (broad coverage) and the use of cross-validation.

Reference	Broad coverage of defect types	Cross-validated
Acikgoz/Korkmaz [19]	✗	✗
Alvarenga et al. [20]	✗	✗
Dang et al. [14]	✗	✗
Li et al. [21]	✗	✗
Lu et al. [22]	✗	✗
Aydin et al. [15]	✗	✓
Hu et al. [16]	✓	✗
Jatoi et al. [17]	✓	✗
Wu et al. [23]	✓	✗
**Our work**	✓	✓

The first group of papers covers only a limited range of defect types, typically addressing fewer than five classes. Ackigoz/Korkmaz [19] develop a residual convolutional network to distinguish healthy samples from joints, squats, and so-called “SSquats”, using 1,838 images and no cross-validation. They report an accuracy of 99.83%. Alvarenga et al. [20] also classify joints and squats, but distinguish them from welding points and achieve a non-cross-validated accuracy of 98.00% based on 2,848 samples. Dang et al. [14] use non-optical sensors and ultrasonic waves to differentiate between intact and damaged rails (with cracks or bumps). The authors achieve an accuracy of 98.00% (900 images, non-cross-validated). Li et al. [24] also use so-called B-Scan ultrasonic images and achieve an accuracy of 85.00% for a problem with more classes (four) but fewer samples (280). Lu et al. [22] again use B-Scan ultrasonic images, but instead of training a CNN, they train a Vision Transformer architecture. This allows global features to be extracted efficiently, resulting in an accuracy of 98.92% for a four-class problem, but also not cross-validated (283 samples). Aydin et al. [15] use 1,598 images to assess fasteners (healthy, damaged, missing) and are able to achieve an accuracy of 99.70%. This work is the only one in the group of studies that does not aim for broad error coverage and that performs cross-validation. Three studies cover a wider range of defect types and therefore use five or more classes. Hu et al. [16] use ultrasonic images to classify eight different degrees of rail defects. The authors use different transfer learning approaches and vision transformers, with a hierarchical classification model showing the highest performance with 88.56% accuracy. Jatoi et al. [17] use the same dataset as the present study (seven classes). As in the work of Hu et al. [16], various vision transformers and transfer learning backbones are tested, but no cross-validation is performed. Using a train-test split, DenseNet121 achieves the highest performance with an accuracy of 92.80%. Finally, Wu et al. [25] use 920 samples in six classes, e.g., different screw cracks, and achieve an accuracy of 97.3%, which is also not cross-validated. Overall, the analysis shows that most studies lack either broad defect coverage or cross-validation and are therefore only suitable to a limited extent for the methodologically and empirically robust development of a model relevant to such a safety-critical application. Aydin et al. [15] do use such validation, but only cover three classes. Models that cover five or more classes are few and do not use cross-validation. Although both broad defect-type coverage and robust evaluation are essential from a practical point of view, existing studies do not meet these requirements simultaneously, leaving a relevant research gap that the present work addresses. Compared with the approaches summarized in Table 1, our method differs in three aspects. First, we address seven different defect classes in a peer-reviewed dataset, thereby offering broader coverage than existing approaches, which typically consider fewer than five types of defects. Second, this study adopts a novel dual-branch architecture that combines CNN-based local feature extraction with a Transformer branch capturing global context, whereas previous studies have mainly relied on single-branch approaches with CNNs. Third, the proposed architecture, DualSightNet, is rigorously validated using stratified 5-fold cross-validation, while most existing works report single train–test splits. Together, these distinctions demonstrate that our approach simultaneously achieves broad defect coverage, hybrid feature extraction, and robust evaluation, criteria that have not been jointly met by any previous study.

## Methodology

This section describes the general methodological approach of this study. It first introduces the proposed DualSightNet architecture and its components, including the ResNet50, Swin Transformer V2, and the Efficient Channel Attention module. This is followed by a description of the training process, the dataset used in this study, and the evaluation metrics applied to assess model performance.

### Model architecture

#### DualSightNet.

**Fig 1:**
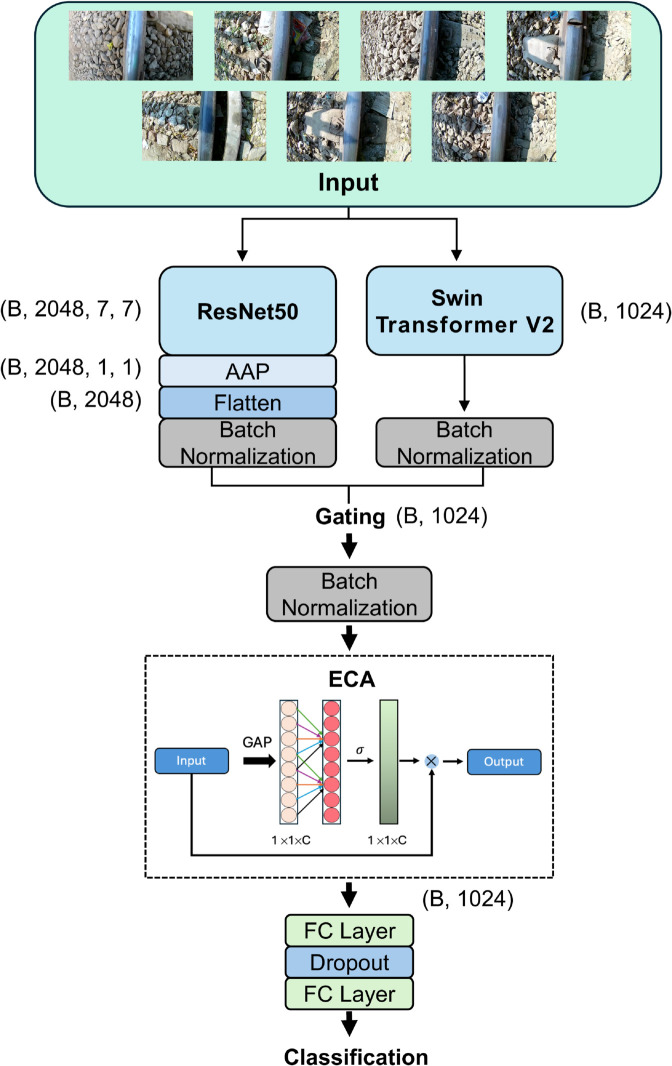
Visualization of DualSightNet. The two architectures, ResNet50 and Swin Transformer V2, each extract key features from the input images of the dataset [18]. From the resulting feature maps, one-dimensional vectors are generated and subsequently fused with the gating mechanism. The fused vector is then processed by the Efficient Channel Attention (ECA) module, which highlights the most relevant features. This enhanced vector is passed through two fully connected layers to produce the final classification output.

The proposed architecture, DualSightNet, integrates convolutional and transformer-based components, leveraging ResNet50 to extract fine-grained local features and Swin Transformer V2 to get more global relationships. This combination is particularly beneficial for the detection of rail defects, as such defects can vary in size, shape, and intensity. The integration of local and global perspectives contributes to the precise classification of these defects. The network is initialized with pretrained ImageNet weights, which promote faster convergence and improved generalization [26]. ResNet50 has been shown to be a robust and scalable backbone [27], with a structural design that helps reduce overfitting [28], thereby enhancing its generalization capabilities [29]. The Swin Transformer V2 enhances DualSightNet by enabling the modeling of global dependencies between local defect features and the broader rail structure. This global contextual understanding is particularly valuable in railway surface defect classification, where small or subtle defects often resemble non-defective components such as the rail bed or surface texture. By integrating global self-attention with local feature extraction from ResNet50, the model is able to distinguish visually similar patterns more accurately and classify defects with higher robustness and precision. The structure of DualSightNet is shown in Fig 1. Both ResNet50 and Swin Transformer V2 independently generate deep feature maps from the input image. ResNet50 produces a feature map (${𝐅}_{\text{ResNet50}}$) of size (B, 2048, 7, 7), whereas Swin Transformer V2 outputs a vector (${𝐟}_{\text{Swin}}$) of size (B, 1024), which is already in a reduced one-dimensional form. The feature map for ResNet50 is then reduced using adaptive average pooling (AAP), resulting in a feature map size of (B, 2048, 1, 1) and then transformed into a one-dimensional vector of size ${𝐟}_{\text{ResNet50}}$ = (B, 2048) by flattening. Eq 1 illustrates this process.

$${𝐟}_{\text{ResNet50}}=\text{Flatten}(\text{AAP}({𝐅}_{\text{ResNet50}}))$$ (1)

Afterward, both one-dimensional vectors are individually passed through batch normalization to stabilize and normalize the intermediate representations [30]. The batch-normalized vectors (${\hat{𝐟}}_{\text{ResNet50}}$ and ${\hat{𝐟}}_{\text{Swin}}$) are then scaled to the same number of channels (1024). This is followed by fusion using a gating mechanism [31], where **g** is a learnable weighting vector:

$$𝐎=g\cdot {\hat{𝐟}}_{\text{ResNet50}}+(1-g)\cdot {\hat{𝐟}}_{\text{Swin}}$$ (2)

In the implementation used, **g** is a channel-wise vector with the same dimensionality as the fused feature representation (1024). It is computed independently for each input sample. The output *O* is then normalized using batch normalization, followed by an ECA attention module, which highlights the most informative channels within the fused feature vector. ECA provides an efficient attention mechanism with minimal computational overhead. The enhanced feature vector, which retains the same dimension, is then passed through two fully connected layers (FC) separated by a dropout layer to mitigate overfitting. The number of neurons in the FC layer is determined by hyperparameter tuning, followed by a ReLU activation and dropout. The second FC layer contains 7 neurons corresponding to the number of classes and produces raw logits without an activation function, as the softmax operation is implicitly applied by the cross-entropy loss during training. This architecture leverages the complementary strengths of the fused features, resulting in a more robust and reliable classifier for complex image classification tasks.

#### ResNet50.

ResNet50 is a deep convolutional neural network [32], introduced in 2016 by He et al. [28,33]. Its design makes it especially effective as a local feature extractor. It emerged as a solution to the degradation problem in deep learning, where adding more layers to a neural network led to higher training error rather than improved performance [28]. ResNet introduced a novel residual learning framework that enabled the construction of very deep networks without the typical optimization difficulties [28]. At its core, ResNet50 employs residual blocks, which reformulate the layers as learning residual functions with reference to the layer inputs, rather than learning unreferenced functions directly [28]. Mathematically, instead of approximating a desired mapping *H*(*x*), ResNet learns a residual function defined as F(x)=H(x) − *x*, making the overall function F(x)  +  *x* easier to optimize [28]. The residual learning approach enables better feature extraction and scalability, making ResNet a cornerstone model in modern deep learning applications [28,33]. The architecture consists of 50 layers, organized into four main components: convolutional layers, identity blocks, convolutional blocks, and fully connected layers. ResNet50 features identity mapping through identity blocks, allowing certain layers to be bypassed when unnecessary, thereby helping to mitigate overfitting [28]. In stages two through five, each contains one convolutional block composed of 1 × 1 convolutions for dimensionality reduction and expansion, and a 3 × 3 convolution for feature extraction. Each convolutional layer is followed by a batch normalization layer. The number of identity blocks varies across stages: stage 2 has two blocks, stage 3 has four blocks, stage 4 has six blocks, and stage 5 has three blocks. Stage 1 consists of a convolutional layer followed by batch normalization, a ReLU activation, and a max pooling layer. For this study, the top layer of the base model is omitted.

#### Swin Transformer V2.

The Swin Transformer, introduced by Liu et al. [34], works by processing images in small, non-overlapping patches and computing self-attention locally within fixed-size windows. Each image is divided into patches that are treated like tokens in a Transformer, and these patches go through several stages [25,34]. The network is composed of four stages, each containing its own Swin Transformer block. Each block includes Layer Normalization (LN), followed by either window-based or shifted window-based multi-head self-attention (W-MSA and SW-MSA), and a multilayer perceptron (MLP) with a GELU activation function. Residual connections are applied throughout the architecture [34]. Between stages, the blocks merge image patches, progressively downsample the feature maps, and increase the feature dimensionality. This structure allows it to extract increasingly abstract features at each stage while keeping computation efficient [34] [25]. A major innovation of Swin Transformer is the shifted window mechanism. In each successive Transformer layer, the windows used for self-attention are shifted slightly, which allows the model to connect and exchange information between neighboring windows. This strategy overcomes the limitation of standard local attention, which can only model relationships within fixed windows [25,34]. The shifting, combined with masking in the attention mechanism, ensures that the model captures broader context across the image while still maintaining computational efficiency [25]. The Swin Transformer’s ability to capture global dependencies is valuable for rail defect detection, as the occurrence and severity of defects often depend on their spatial context within the rail structure. By capturing global relationships and integrating information across the entire image, the model can more accurately detect and distinguish defects of varying sizes and types, even in complex environments. This makes it well-suited for robust defect detection.

#### Efficient channel attention.

**Fig 2:**
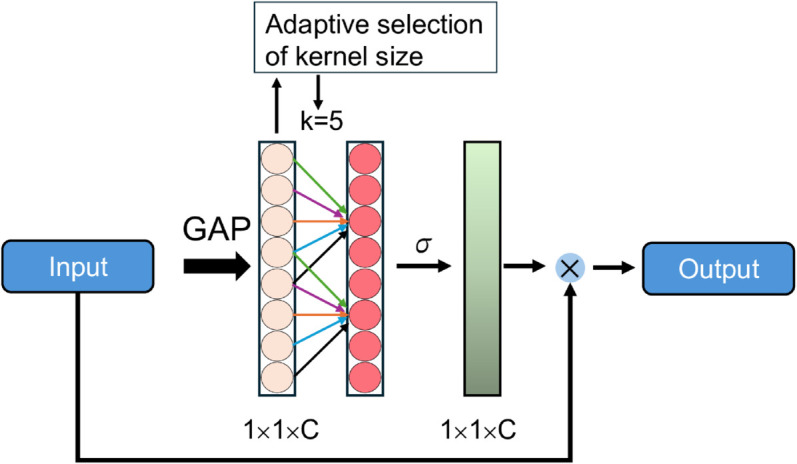
Schematic representation of efficient channel attention (ECA) [35].

Efficient Channel Attention (ECA), shown in Fig 2, is a lightweight attention module designed to selectively emphasize the most relevant channels in a feature map. Compared to the earlier Squeeze-and-Excitation (SE) module, ECA introduces several improvements. Unlike SE, which relies on complex non-linear transformations and dimensionality reduction, ECA avoids such operations by applying a simple 1D convolution without reducing channel dimensions, making it both efficient and parameter-light [35]. The process begins with Global Average Pooling (GAP), which produces a 1D vector. This vector is then passed through a 1D convolutional layer with an odd kernel size *k*. The kernel size is not fixed but adaptively determined based on the number of channels *C* as follows [35]:

$$k=\psi (C)={\left|\frac{\log_{2}(C)}{\gamma }+\frac{b}{\gamma }\right|}_{\text{odd}}$$ (3)

This method maintains both effectiveness and efficiency. While the SE block relies on computationally expensive fully connected layers, ECA achieves similar performance with significantly lower overhead by using a lightweight 1D convolution with an adaptively determined kernel size [35]. *γ* represents the scale factor and *b* the offset. Finally, the result is rounded to the nearest odd number [35]. To emphasize the most important feature channels, the resulting vector is multiplied element-wise with the original feature map. [35]. As a lightweight module, ECA effectively highlights the most relevant features. This is especially beneficial after feature fusion, where global representations from the Swin Transformer V2 are combined with local features from ResNet50. In this context, ECA enables the model to automatically identify and enhance the most informative channels within the fused representation.

### Process of training

**Fig 3:**
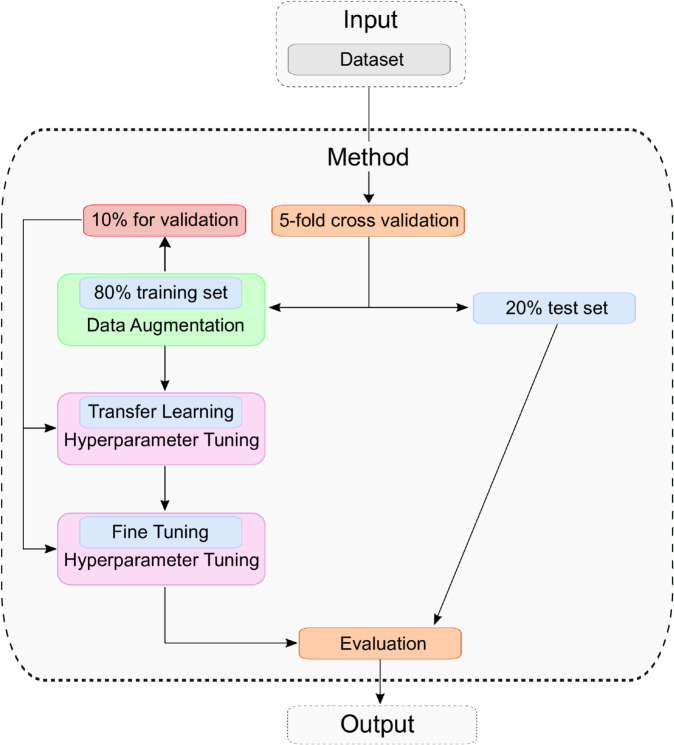
Training and Evaluation approach: The dataset is split into a training set and a test set. Data augmentation is applied to the training set, followed by model training using transfer learning and fine-tuning. Finally, the resulting model is evaluated on the test set.

The entire training process is shown in Fig 3. For training and testing the architecture, an NVIDIA L40S GPU with 48 GB memory and PyTorch 2.5.0 were used. Python version 3.11.7 and CUDA 12.4.1 were also employed. Before model training, a stratified 5-fold cross-validation was applied using the scikit-learn library [36] (version 1.5.2) to divide the dataset into five equally sized subsets. This method was chosen to ensure that each fold maintains the original class distribution, unlike standard k-fold cross-validation, which may produce biased results due to class imbalance. In each iteration, four folds (80%) are used for training, and one fold (20%) is held out for testing. This process is repeated five times, rotating the test fold each time to ensure robust performance evaluation. Within the training set of each fold, 10% is further reserved for validation and hyperparameter tuning. This proportion yielded better results in preliminary tests than validation splits of 15% and 20%. Note that the validation data is only used for hyperparameter tuning and not for training or testing. Using a higher percentage, therefore, reduces the training dataset, which could explain the worse results. No data augmentation techniques or other image enhancement methods were applied before the training process. The original images from the dataset were used. All images are resized to 224 × 224 pixels with three RGB channels and normalized using the ImageNet mean and standard deviation. To address the imbalance between the individual defect classes, the class weights are integrated into the loss function (CrossEntropy(weight=class_weights)) in order to also take rarer classes into account. To reduce overfitting and improve generalization, the training data is augmented using RandomRotation(degrees=10), RandomResizedCrop(scale=(0.9, 1.0)) and RandomAffine(degrees=0, translate=(0.1, 0.1). These transformations account for variability in image scale and position, making the model more robust to input variations. All transformations are applied on-the-fly during training. These techniques are only applied to the training data. The validation and test data are not augmented. Each model is trained for a maximum of 100 epochs with a batch size of 16. The training consists of two phases: transfer learning and fine-tuning. A batch size of 16 was used during transfer learning to maintain stable optimization and ensure comparability between runs, since only the classification head was updated at this stage. This size offered a practical balance because smaller batches can produce noisier gradients, while larger ones increase memory consumption. During fine-tuning, when all layers were unfrozen, batch sizes of 8, 16, and 32 were tested to examine their influence on convergence and generalization. During transfer learning, the pretrained feature extraction layers (based on ImageNet weights [26]) are frozen, while the fully connected layers remain trainable. In the fine-tuning phase, the entire model is updated. Hyperparameter optimization is performed separately for both training phases using Optuna (version 4.2.1) with a Tree-Structured Parzen Estimator (TPE) approach, testing the following parameters: dropout, learning rate, weight decay, number of units in the first fully connected layer, batch size, and optimizer. Some parameters are only tested in transfer learning or fine-tuning. An overview of these parameters with the corresponding search interval can be found in Table 2; no other parameters are tested. For each fold, 20 trials are conducted, and the configuration that yields the lowest validation loss is selected. Each trial is allowed to run up to 100 epochs. To avoid overfitting and reduce computational overhead, early stopping (patience=10, min_delta=0.001) is implemented, terminating training if the validation loss does not decrease over ten consecutive epochs. After identifying the optimal hyperparameters, a final transfer learning model is trained using the same configuration as during optimization. The model with the lowest validation loss is saved and used as the base for the subsequent fine-tuning phase. In fine-tuning, the hyperparameters (see Table 2) are re-optimized, and additional feature extraction layers are unfrozen to enable further domain adaptation. The hyperparameter tuning now again includes dropout, learning rate, weight decay, and the number of units in the first fully connected layer, and additionally incorporates the batch size. Again, the best-performing model based on validation loss is stored. After training, the final model is evaluated on the 20% test set using the following performance indicators such as Accuracy (Eq 4), Balanced accuracy (Eq 5), True positive rate (Eq 7) (sensitivity or recall), True negative rate (Eq 8) (Specificity), Positive predictive value (Eq 9) (precision), Negative predictive value (Eq 10), Cohen’s Kappa (Eq 6), and F1-Score (Eq 11). These metrics are computed for each fold, stored, and finally averaged across all five folds to provide a comprehensive overview of model performance. The training time for DualSightNet was 14 hours and 39 minutes, whereas the baseline models required less time for comparison, with 4 hours and 25 minutes for ResNet50 and 12 hours and 48 minutes for Swin Transformer V2.

**Table 2:** Overview of the hyperparameters used for hyperparameter tuning. TL = Transfer learning; FT = Fine-Tuning.

Hyperparameter	Minimum Value	Maximum Value	Step	TL or FT
Dropout	0.1	0.5	0.05	TL, FT
Learning Rate (1)	10^−4^	10^−2^	calculated logarithmically	TL
Learning Rate (2)	10^−6^	10^−4^	calculated logarithmically	FT
Weight decay	10^−5^	10^−3^	calculated logarithmically	TL, FT
1. FC layer units	128	1024	128	TL, FT
Batch size	-	-	8, 16 or 32	FT
Optimizer	-	-	AdamW, SGD	TL

### Dataset

**Fig 4:**
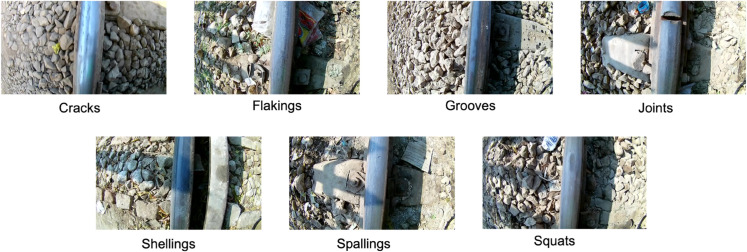
Overview of example images showing defects present in the dataset [18].

This study makes use of the Railway Track Surface Faults Dataset by Arain et al. [18], which offers a comprehensive and diverse set of images designed for the detection and classification of different defects on tracks and is available under DOI:10.17632/8hxtgyyxrw.2. The dataset was chosen because its seven defect classes capture most defect types encountered in real railway environments, and the images were recorded under authentic operational conditions. The resulting data include natural noise, shadows, and variations in the ballast bed, creating realistic variability that supports robust model generalization. These properties make the dataset well-suited for training and evaluating reliable railway defect-classification models. Data collection took place at Kotri Junction, Pakistan, one of the busiest sections of the national railway network. Two EKEN H9R cameras were mounted on a railway inspection vehicle, each operating at 120 frames per second with a field of view of approximately 14 inches. To ensure sufficient image detail, the inspection vehicle maintained a controlled speed of 20 km/h throughout recording. Based on the available images, the data was collected during the day. Although there are no signs of adverse weather conditions such as rain, precise weather conditions cannot be determined. After data collection, the recorded video material was pre-processed to remove irrelevant sections, followed by frame extraction and manual annotation. No further information about subsequent data augmentation or image enhancement is mentioned. The dataset contains a total of 5,153 images with a resolution of 1280 × 720 px, divided into seven defect categories as can be seen in Fig 4: Cracks, Flakings, Grooves, Joints, Shellings, Spallings, and Squats. The respective number of individual classes can be found in Table 3. The defects occurred naturally, with some stemming from actual operational incidents, thus providing authentic examples for research.

**Table 3:** Overview of the seven defect classes with the number of images per class used in this study.

Cracks	Flakings	Joints	Shellings	Spallings	Squats	Grooves
40	2,829	11	130	291	1,844	8

### Evaluation metrics

We employ multiple performance metrics to evaluate the effectiveness of the proposed model. These include *accuracy*, *kappa*, *true positive rate*, *true negative rate*, *balanced accuracy*, *positive predictive value*, *negative predictive value*, *F1-score*. The following metrics refer to the multi-class case, taking into account the class weighting, *n*_*i*_ represents the number of samples per class and *S* denotes the total number of samples. The following provides a brief explanation of each metric:

1. **Accuracy**
  Accuracy [37] reflects the overall proportion of correct predictions made by the model. It is defined as:
  $$\text{Accuracy}=\frac{\text{TP}+\text{TN}}{\text{TP}+\text{TN}+\text{FP}+\text{FN}}$$ (4)
  where *TP*, *TN*, *FP* and *FN* denote true positives, true negatives, false positives and false negatives, respectively.
2. **Balanced Accuracy**
  Balanced accuracy is particularly useful when dealing with imbalanced class distributions. In multiclass settings, as addressed in this study, it is computed as the average of the TPRs across all classes [38]. *N* denotes the number of classes.
  $$\text{Balanced Accuracy}=\frac{1}{N}\sum_{i=1}^{N}\frac{TP_{i}}{TP_{i}+FN_{i}}$$ (5)
3. **Kappa**
  Cohen’s kappa [39] (κ) measures the agreement between predicted and actual classifications, adjusted for the agreement that might occur by chance. It ranges from –1 (complete disagreement) to 1 (perfect agreement):
  $$\kappa =\frac{P_{o}-P_{e}}{1-P_{e}}$$ (6)
  where *P*_*o*_ is the observed agreement and *P*_*e*_ the expected agreement by chance.
4. **True Positive Rate (TPR)**
  Also known as recall or sensitivity, TPR indicates the model’s ability to correctly identify positive cases [40]. TPR is calculated by [41]:
  $$\text{TPR}=\sum_{i=1}^{N}\frac{n_{i}}{S}\cdot \frac{TP_{i}}{TP_{i}+FN_{i}}$$ (7)
5. **True Negative Rate (TNR)**
  TNR, also known as specificity, is analogous to the TPR, but for the negative class. It measures how effectively actual negative instances are correctly classified as negative [42]:
  $$\text{TNR}=\sum_{i=1}^{N}\frac{n_{i}}{S}\cdot \frac{TN_{i}}{TN_{i}+FP_{i}}$$ (8)
6. **Positive Predictive Value (PPV)**
  PPV [43], also known as precision, quantifies the proportion of positive predictions that are actually correct:
  $$\text{PPV}=\sum_{i=1}^{N}\frac{n_{i}}{S}\cdot \frac{TP_{i}}{TP_{i}+FP_{i}}$$ (9)
7. **Negative Predictive Value (NPV)**
  In contrast to the PPV, the NPV indicates the proportion of true negative classifications among all instances that were predicted as negative [44]:
  $$\text{NPV}=\sum_{i=1}^{N}\frac{n_{i}}{S}\cdot \frac{TN_{i}}{TN_{i}+FN_{i}}$$ (10)
8. **F1-Score**
  The F1-score [37] represents the harmonic mean of precision (PPV) and recall (TPR), balancing both metrics in a single value. It is particularly useful for evaluating models on imbalanced datasets. F1 is calculated as follows for the multiclass case [45]:
  $$\text{F1-Score}=\sum_{i=1}^{N}\frac{n_{i}}{S}\;2\cdot \frac{PPV_{i}\cdot TPR_{i}}{PPV_{i}+TPR_{i}}$$ (11)

## Results and discussion

In this section, we present the results achieved by the proposed architecture, DualSightNet, and discuss their broader implications. Table 4 summarizes the classification performance across all five folds as well as the overall average and standard deviation.

**Table 4:** Performance of DualSightNet over five cross-validation runs. All metrics are reported as percentages, except for the Kappa value. The average values (AVG) are displayed at the end of the table with the corresponding standard deviation (SD).

Fold	1	2	3	4	5	AVG ± SD
Accuracy	97.28	96.12	96.41	96.21	96.60	**96.53 ± 0.46**
Bal. Acc.	98.34	96.22	96.87	97.92	98.38	**97.55 ± 0.96**
TPR	97.28	96.12	96.41	96.21	96.60	**96.53 ± 0.46**
TNR	98.65	98.39	98.60	97.43	97.73	**98.16 ± 0.55**
PPV	97.39	96.76	96.72	96.35	96.75	**96.79 ± 0.37**
NPV	97.52	96.65	96.77	96.94	97.51	**97.08 ± 0.41**
Kappa	0.9525	0.9325	0.9374	0.9335	0.9403	**0.9392 ± 0.0080**
F1-Score	97.31	96.27	96.48	96.25	96.63	**96.59 ± 0.43**

**Fig 5:**
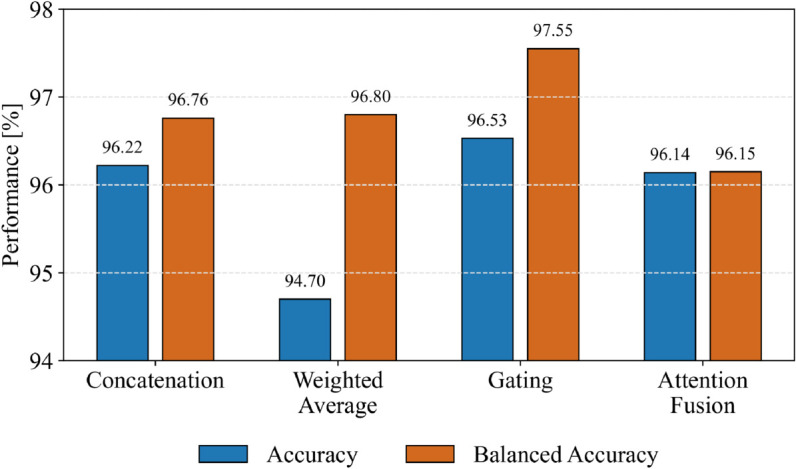
Bar chart illustrating the accuracy (blue) and balanced accuracy (orange) achieved by DualSightNet with different fusion mechanisms. Fusion mechanism gating achieved the highest values.

The model was evaluated using stratified 5-fold cross-validation. The results show that the model achieves an accuracy (see Eq 4) ranging from 96.12% to 97.28%, with an overall average of 96.53%. In terms of balanced accuracy (Eq 5), which is important for imbalanced classes, the architecture achieves values between 96.22% and 98.38%, averaging 97.55%. On average, the TPR (Eq 7) reaches 96.53%, PPV (Eq 9) reaches 96.79%, and F1-score (Eq 11) 96.59%, indicating a high level of sensitivity and precision across classes. Even higher values are achieved for the TNR (Eq 8), with an average of 98.16%, while the NPV (Eq 10) also reaches a high value of 97.08%. These results imply that the model is highly reliable in rejecting instances that do not belong to a given class, with minimal confusion between different categories. A Cohen’s Kappa (Eq 6) score of 0.9392, indicating strong agreement between predicted and actual defect categories. Various fusion mechanisms were tested for evaluation purposes. The results achieved for accuracy and balanced accuracy can be seen in Fig 5, with gating fusion mechanisms achieving the highest values.

DualSightNet with gating achieves a balanced accuracy that is 0.79 percentage points (pp) higher than the second-best result, with an increase in accuracy of 0.31 pp. In addition, a study was conducted to evaluate the effectiveness of the individual components and overall compared to other DL architectures. The results are shown in Table 5. It can be seen that DualSightNet achieves the best result in terms of balanced accuracy. Compared to baseline models, DualSightNet achieves an increase of: VGG19 (+7.80 pp), MobileNetV2 (+4.12 pp), EfficientNet-B4 (+6.01 pp), ResNet50 (+1.99 pp), and Swin Transformer V2 (+1.63 pp). Furthermore, a Wilcoxon signed-rank test was performed to demonstrate the significance of DualSightNet and other DL models: VGG19 (p = 0.0313), MobileNetV2 (p = 0.0313), EfficientNet-B4 (p = 0.0313), ResNet50 (p = 0.2188), and Swin Transformer V2 (p = 0.1563). This indicates that the separate fold results of DualSightNet are statistically significant compared to those of VGG19, MobileNetV2, and EfficientNet-B4. DualSightNet uses two baseline architectures to achieve particularly high performance, which is also reflected in the number of parameters. It has the following trainable parameters per fold: 116,466,763 (fold 1); 118,047,819 (fold 2); 118,443,083 (fold 3); 118,047,819 (fold 4), and 117,652,555 (fold 5). ResNet50, on the other hand, has the following trainable parameters per fold: 25,095,239 (fold 1); 25,095,239 (fold 2); 23,779,399 (fold 3); 23,779,399 (fold 4), and 25,621,575 (fold 5). The number of trainable parameters per fold of Swin Transformer v2 is as follows: 87,964,671 (fold 1); 87,039,999 (fold 2); 87,700,479 (fold 3); 87,964,671 (fold 4) and 87,700,479 (fold 5).

**Table 5:** Comparison of the individual components of DualSightNet (DSN) and other deep learning architectures. The table reports the balanced accuracy for each fold of the stratified 5-fold cross-validation as well as the mean and standard deviation across all runs. The ablation isolates the contributions of the ResNet50 branch, the SwinV2 branch, and the ECA module to the overall performance.

Fold	1	2	3	4	5	AVG
VGG19	97.33	95.03	85.22	90.46	80.70	**89.75**
MobileNetV2	94.40	95.79	90.99	96.82	89.14	**93.43**
EfficientNet-B4	96.02	98.32	85.73	96.72	90.04	**91.54**
ResNet50	96.17	98.41	93.52	99.03	90.67	**95.56**
SwinV2	97.75	96.67	96.56	97.55	91.07	**95.92**
DSN (no ECA)	98.35	98.77	92.93	98.49	97.02	**97.11**
DSN	98.34	96.22	96.87	97.92	98.38	**97.55**

**Fig 6:**
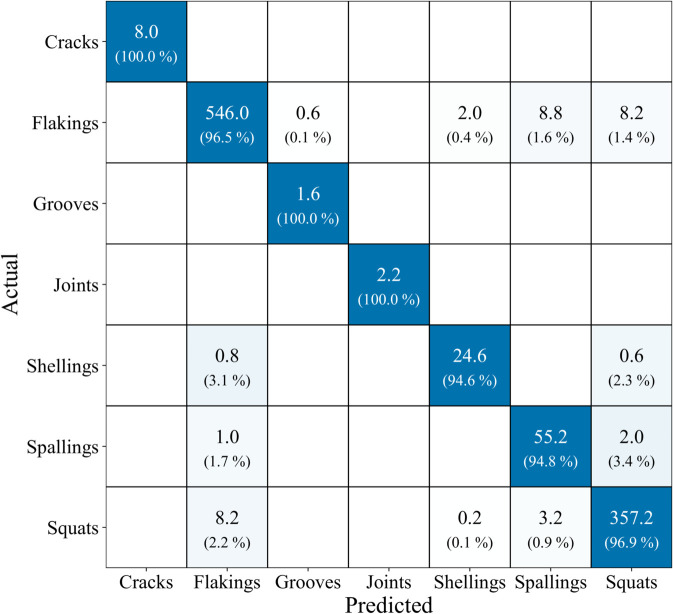
Average confusion matrix across all five folds. Absolute counts per class pair are shown; the percentages underneath each value denote the share of that cell relative to the total number of samples in the corresponding actual class (row-normalized).

In addition to the performance metrics, Fig 6 presents the average confusion matrix. This figure shows the absolute classification values aggregated across all five folds, rounded to two decimal places. Below each absolute value, the corresponding row-normalized percentage is displayed.

**Fig 7:**
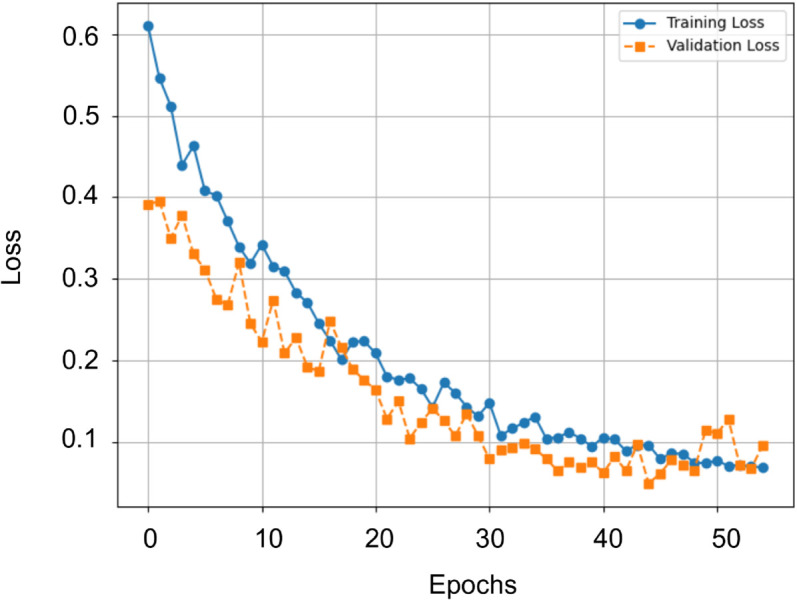
Illustration of a training and validation loss curve during the training process. The process was terminated by early stopping.

The classes Cracks, Grooves, and Joints were correctly classified in 100.00% of the cases. The classes Flakings and Squats were classified with over 96% accuracy, showing few misclassifications on average. Spallings and Shellings followed closely with 94.85% and 94.62% accuracy, respectively. As the matrix shows, the three underrepresented classes (Cracks, Grooves, and Joints) are recognized at 100%, indicating no systematic disadvantage due to the number of images. The Shellings and Spallings classes, which also have lower three-digit sample counts, achieve a recognition accuracy comparable to the two largest classes. This demonstrates that DualSightNet can deliver strong recognition performance even under severe class imbalance, which is a valuable property for practical applications where rare defect types may occur with only a few samples available. The confusion matrix also shows that the most frequent mix-ups occur between visually similar defect types. The most common errors are spallings that are classified as squats (3.4% of spalling samples) and shellings that are classified as flakings (3.1% of all shelling samples). This pattern is consistent with the visual characteristics of these groups. Both spallings and squats demonstrate large-scale material changes, but these do not have a clear visual form, such as joints. Shellings, on the other hand, represent small-scale material breakouts that closely resemble the superficial chipping observed in flakings. The defect pattern, therefore, reflects the visual similarity of the categories and indicates that the model follows real-world visual relationships. Fig 7 shows the training and validation loss for one fold. They demonstrate stable training behavior and no signs of overfitting. The training process was stopped by the early stopping mechanism because the validation loss did not decrease for 10 epochs.

**Fig 8:**
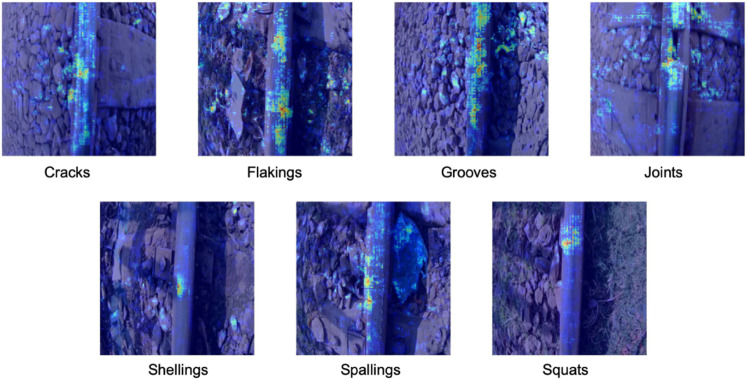
Exemplary occlusion maps for one sample per class, illustrating that the model consistently focuses on the structurally relevant regions associated with the damage patterns of each category.

The explainability of AI models is particularly important in the field of infrastructure monitoring. To this end, a systematic occlusion-based analysis was performed in which image areas were systematically obscured and the changes in the model’s predictions were quantified and visualized. This allows to show how strongly local structures contribute to the model’s decision. Fig 8 shows the results of this evaluation for one sample per class. It can be seen that DualSightNet always accurately focuses on the relevant sections of the rails: for cracks and grooves on longer sections of the rails, for joints at the corresponding beginning of the rail opening, and for shellings, spallings and squats, which represent more local damage, at the corresponding damaged areas. On the one hand, this shows that the model correctly learns real-world features; on the other hand, the analysis illustrates the potential of explainable AI for the acceptance of such systems in practice.

### Interpretation of the results

**Fig 9:**
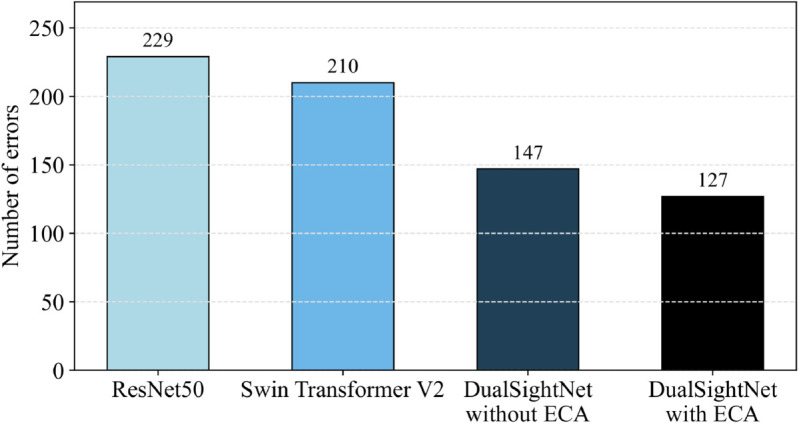
Representation of the absolute errors of the respective architectures. DualSightNet with ECA achieves the best value.

The effectiveness of DualSightNet with ECA is illustrated in Fig 9. This figure shows the absolute number of misclassifications for each architecture: ResNet50, Swin Transformer V2, DualSightNet without ECA, and DualSightNet with ECA. A clear reduction can be seen here, starting with ResNet50, which has the most errors with 229, followed by Swin Transformer V2 with 210. A more substantial improvement appears with DualSightNet without ECA, which has 147 errors. This number is further reduced by DualSightNet with ECA, which achieves the lowest overall error count. Compared to the other architectures, this results in 102 (ResNet50), 83 (Swin Transformer V2), and 20 fewer errors. In other words, the proposed DualSightNet manages to reduce errors by 44.54% (ResNet50), 39.52% (Swin Transformer V2), and 13.61% (DualSightNet without ECA). This result is of great significance in quality control and in the detection of rail defects. Certain defects on the rail can be safety-relevant depending on their severity. If these defects are not detected or are incorrectly classified, the train traveling on that rail can ultimately be endangered, putting human lives at risk. The proposed architecture reduces unnecessary follow-up investigations by reducing classification errors. Thanks to more accurate classification, maintenance can be carried out earlier and more reliably. Safety-critical areas can also be prioritized more precisely. As a result, personnel and equipment can be deployed more efficiently, reducing costs and making more effective use of human resources. In summary, DualSightNet helps improve the quality of defect detection and, consequently, safety.

Although some classes are severely underrepresented in the dataset, such as cracks (40 images), joints (11), or grooves (8), and other classes such as flakings (2,829) and squats (1,844) are heavily overrepresented, the results of the confusion matrix (Fig 6) show that the model can still reliably detect these underrepresented defects. This indicates that the weighted loss function and the architecture can effectively learn and recognize minority classes. However, there remains a certain risk arising from the limited number of real training examples. Even if the balanced accuracy is high, it is based on the few images available for the underrepresented classes. This means that variations in the images or new forms of these defects during real operation may not have been fully covered by the training data. As a result, model performance for these rare defects may fluctuate more and have lower generalization ability under real-world operating conditions than for classes with many training examples, such as spalling or squats, especially for new, previously unknown variants of the defects.

When comparing DualSightNet with relevant studies, two aspects stand out in particular. A significant proportion of studies do not evaluate their models using robust methods such as cross-validation [14,16,17,19,20,22,24,25]. The use of train-test splits can obscure the variability of model performance, as, for example, a particularly advantageous split may be used for validation, falsely suggesting high overall model performance. Our approach uses stratified 5-fold cross-validation and provides a more reliable robustness estimation, better meeting the requirements of safety-critical railway inspection. Only in [15] is cross-validation used, whereby the focus here is on three fastener states (healthy, damaged, missing). Furthermore, the fact that many approaches focus on fewer than five defect classes limits their use in real-world inspection conditions, as it means that many defects are overlooked. In our approach, we use a dataset that contains seven different types of errors recorded under realistic environmental conditions. This achieves broader defect coverage, which is important for inspections. Jatoi et al. [17] also used the same dataset, enabling us to compare our study with it most closely. However, in a direct comparison, they did not use hyperparameter tuning or cross-validation. They also employed data augmentation techniques to artificially expand the dataset. Their best model, DenseNet121, achieved a test accuracy of 92.80%. In contrast, we achieved an average accuracy of 96.53% and a balanced accuracy of 97.55%, both of which are cross-validated.

### Implications of the study

The findings of this work indicate that deep learning-based image classification can serve as a valuable component in the automation of railway surface defect detection. The strong performance of the proposed architecture underlines its practical relevance and makes it a suitable candidate for integration into a larger defect-classification system. A key question for future implementation is the optimal location for deploying such a system. One possibility is to integrate it directly into passenger or freight trains, enabling continuous monitoring during regular operation. However, it is essential to first assess whether such an installation is technically feasible. Trains are often closed systems, and retrofitting them, provided that space is available, requires thorough evaluation to ensure that the deep learning system does not interfere with existing onboard technologies. Moreover, sufficient computational resources must be available to allow for fast and reliable classification. These are critical factors, especially if the system is to be integrated into regular passenger or freight trains, as no external monitoring vehicles may be available. In such cases, the system must function without altering existing operational conditions such as train speed. Due to these constraints, this option appears to be less suitable for practical deployment. A more practical solution is the use of a mobile platform, such as an inspection vehicle. Since such vehicles are not intended for passenger or freight transport, safety-critical retrofitting requirements are likely to be less restrictive. Sufficient computing resources are still required for reliable defect detection. One major advantage of inspection vehicles is their ability to adjust speed. If, for example, real-time detection at 150 km/h is not feasible due to limited processing capacity, these vehicles can reduce their speed to ensure optimal inspection performance. Another possible solution is the implementation of the system on drones. These can fly along railway lines and assess the condition of track sections from above. However, this method has certain limitations, including a restricted range, limited battery life, and potential issues with weather conditions that may hinder flight operations. The hybrid approach used in this study achieves an average inference time of 3.00 ms per image while operating at 21.20 GFLOPs. Although the hybrid architecture has higher computational costs and longer inference time compared to simpler architectures, such as ResNet50 (1.33 ms/img, 4.11 GFLOPs) and Swin Transformer V2 (2.48 ms/img, 17.08 GFLOPs), it achieves increased balanced accuracy in a safety-critical area. In practical applications, the extent to which longer inference times or higher computing costs are critical should be examined. Switching to simpler models may result in a decrease in recognition accuracy, which, in the worst case, could endanger human lives.

### Limitations

This study introduced DualSightNet, a novel and high-performing hybrid deep learning architecture. Although the results highlight the effectiveness and robustness of the proposed approach, certain limitations of the work should be acknowledged. The dataset employed in this study includes seven predefined defect classes, which means the strong performance of the proposed architecture is currently limited to this specific set of defects. In practice, however, additional and potentially unforeseen defects may appear on railway tracks, and the detection of such defects cannot be evaluated with this dataset. Consequently, the model’s ability to generalize to a wider variety of defects remains uncertain. Furthermore, since the dataset does not include a wide range of weather conditions, no conclusions can be drawn about the robustness of DualSightNet under such varying environmental factors.

### Future work

Building on the outlined limitations, several directions for future research emerge. To enhance the external validity and generalizability of DualSightNet, future studies should aim to evaluate the model on additional datasets that contain not only different imaging conditions but also new defect classes not present in the current dataset. Such evaluations would allow for a more comprehensive understanding of the model’s robustness across varying scenarios. Furthermore, future research could address the current limitations related to the narrow range of environmental conditions, such as weather variations or different times of day, by leveraging generative approaches. Generative Adversarial Networks or diffusion models offer promising tools for this purpose, as they can be used to augment existing datasets with realistic synthetic variations that reflect diverse real-world conditions. Additionally, generative models can be employed to tackle dataset imbalance by producing representative samples for underrepresented defect types or rare scenarios, ultimately contributing to more balanced and effective model training.

## Conclusion

This work presents DualSightNet, a hybrid architecture that integrates CNN-based local feature extraction with transformer-based global representation learning. The model introduces a gating fusion strategy and an efficient channel attention module, and it demonstrates the importance of these elements. With an average balanced accuracy of 97.55%, supported by a TNR of 98.16% and a PPV of 96.79%, DualSightNet establishes a new benchmark for the classification of railway surface defects. The use of stratified 5-fold cross-validation demonstrates the robustness of the model and confirms its ability to reliably detect both common and rare defect types, even under class imbalance. Taken together, these results show that hybrid architectures can address limitations of single deep learning models and offer a validated path toward more dependable, large-scale rail quality-assurance systems.
